# The Efficacy and Safety of <2 cm Micro-Keyhole Microvascular Decompression for Hemifacial Spasm

**DOI:** 10.3389/fsurg.2021.685155

**Published:** 2021-07-28

**Authors:** Jiashang Huang, Yan Zhan, Yi Li, Li Jiang, Kuan Wang, Zhimin Wu, Yanfeng Xie, Quanhong Shi

**Affiliations:** ^1^Department of Neurosurgery, The First Affiliated Hospital of Chongqing Medical University, Chongqing, China; ^2^Department of Rehabilitation Medicine, The First Affiliated Hospital of Chongqing Medical University, Chongqing, China

**Keywords:** micro- keyhole, microvascular decompression, hemifacial spasm, complications, hearing loss, cerebrospinal fluid leakage

## Abstract

**Objective:** Microvascular decompression (MVD) surgery has been accepted as a minimally invasive surgical modality for the treatment of hemifacial spasm (HFS). However, the size of the bone window does not match the concept of minimally invasive. This study is aimed at evaluating the efficacy and safety of <2 cm micro-keyhole MVD.

**Methods:** A total of 148 patients with HFS diagnosed in the First Affiliated Hospital of Chongqing Medical University from January 1, 2019, to July 1, 2020, who underwent MVD in the neurosurgery department of the hospital were collected. Surgery was performed by a retrosigmoid keyhole approach with the bone hole diameter <2 cm, which was named micro-keyhole MVD. The efficacy and safety of the micro-keyhole MVD were evaluated by statistical analysis of the efficacy of the micro-keyhole MVD and the incidence of postoperative complications.

**Results:** The effect of micro-keyhole MVD was satisfying (cure or partial remission) in 97.2% (*n* = 144). The failure and recurrence rates were 2.7% (*n* = 4) and 0.6% (*n* = 1), respectively. Among them, immediate facial palsy, delayed facial palsy, hearing loss, and cerebrospinal fluid (CSF) leakage were found in 0.6% (*n* = 1), 8.1% (*n* = 12), 4.7% (*n* = 7), and 1.3% (*n* = 2). Only one patient developed cerebellar infarction, which was complicated by “moyamoya disease.” The micro-keyhole MVD in the treatment of HFS can achieve a high remission rate and reduce the incidence of surgical complications.

**Conclusion:** Micro-keyhole MVD is a safe and effective minimally invasive treatment for HFS. This technique does not increase the incidence of cranial nerve injury. Meanwhile, it reduces the incidence of CSF leakage and hearing loss (HL).

## Introduction

For primary hemifacial spasm (HFS), the most commonly proposed etiology is neurovascular compression (NVC) at the root exit zone (REZ) of the facial nerve (1). The efficacy of microvascular decompression (MVD) in the treatment of HFS has been confirmed, and the effective remission rate of MVD ranged from 90.5 to 92.8% (2–6). Proposed in 1962 and described detailedly in 1999 (7), MVD had gradually become a wild-accepted treatment for HFS. The diameter of the bone window of MVD was about 4–5 cm, and it was designed as a triangle. In the first 10 years of the 20th century, with the advancement of technology, the size of the bone window gradually decreased to 3 cm (8). In the past 10 years, due to the development of endoscopic technology, the bone window of MVD was more minimally invasive, and the diameter has reduced to <2 cm (9–11). As the bone window decreases, the incidence of cerebrospinal fluid (CSF) leakage in patients is getting lower and lower. However, endoscopes are not configurable in every medical center. In this series of cases, using a microscope, the bone window is designed to be more minimally invasive (<2cm), and we call it micro-keyhole MVD.

## Methods

### Population

We retrospectively reviewed the medical records of 148 patients who underwent micro-keyhole MVD *via* a retrosigmoid keyhole approach between January 2019 and July 2020. All surgeries were performed by a single surgeon (Dr. Yan Zhan) at the First Affiliated Hospital of Chongqing Medical University. All patients were routinely examined by magnetic resonance imaging (MRI) to exclude posterior fossa tumors. Table 1 shows the general characteristics of the patient population.

**Table 1:** General characteristics of the patient population.

	***N* = 148**
Sex, male/female, *n* (ratio)	58:90 (0.64:1)
Age at surgery, year median (range)	51 (21–71)
Side of operation: left /right, *n* (ratio)	76:72 (1:0.94)
Preop duration of symptom, year median(range)	4 (0.25–25)
Follow-up period, months	≥6

### Operative Technique

**Figure 1:**
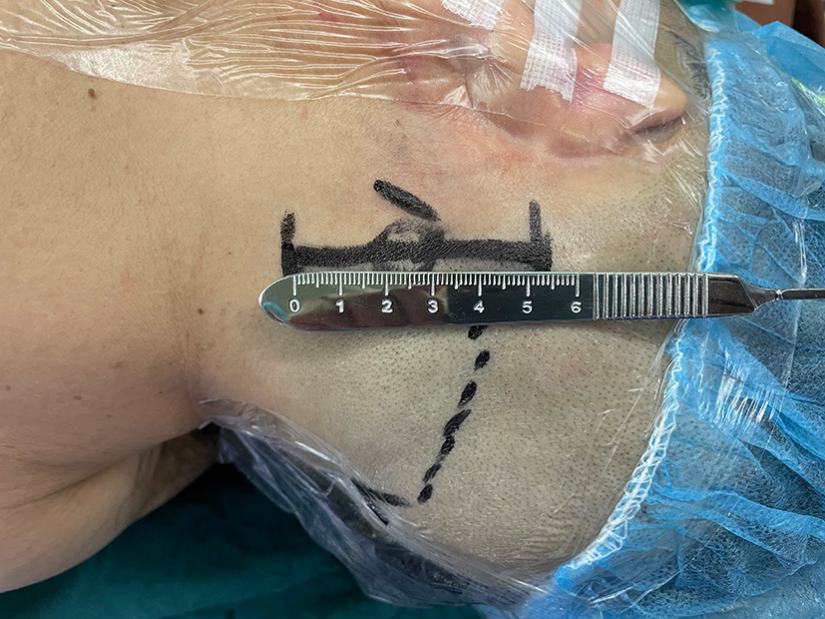
Skin incision in the retroauricular region, with a length of about 5 cm.

**Figure 2:**
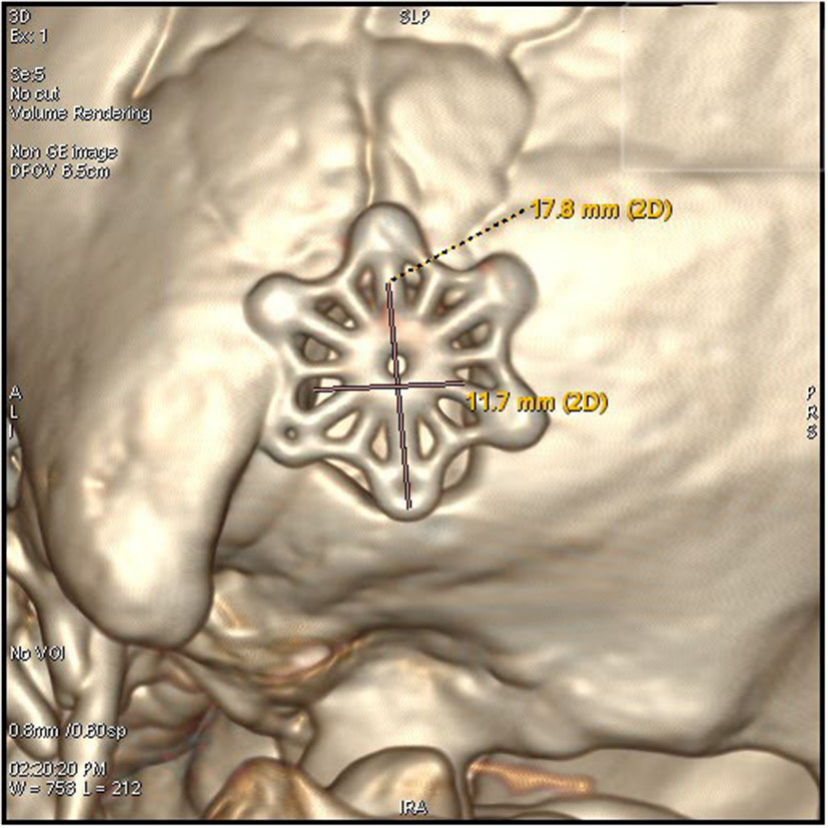
The keyhole craniectomy (17.8*11.7 mm window), an external view of the post-operative 3D reconstruction of the bone window.

**Figure 3:**
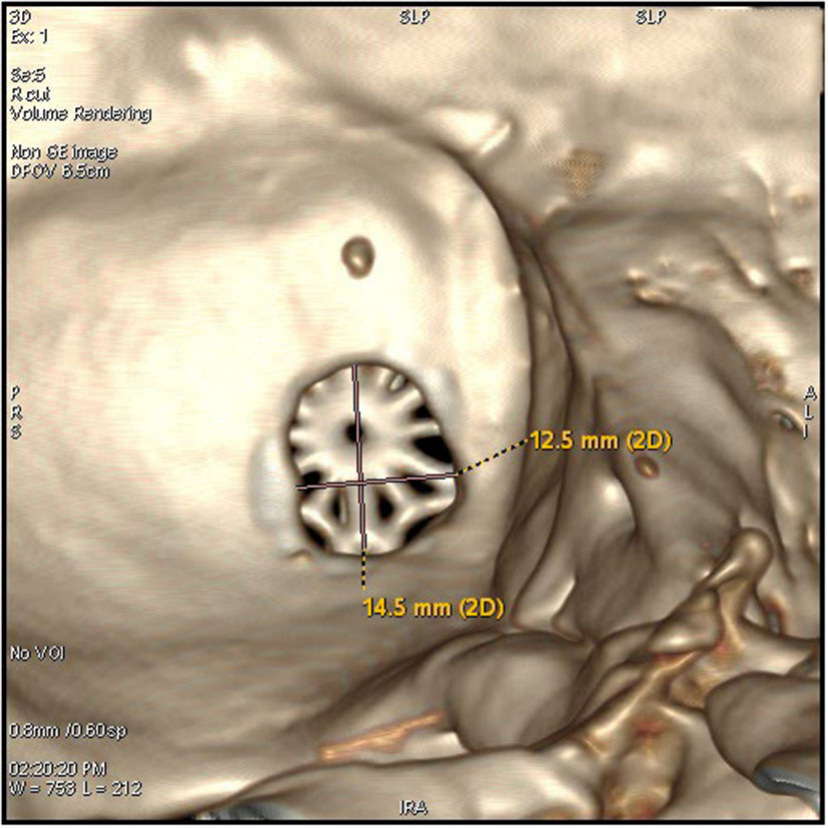
The keyhole craniectomy (14.5*12.5 mm window), an internal view of the post-operative 3D reconstruction of the bone window.

1. Under general anesthesia, the patient was placed in a lateral decubitus position with the head facing the healthy side. With the help of two fixation bands, the patient was fixed to the operating table. During the operation, the head position could be adjusted freely by tilting the operating table to the left or right.
2. The longitudinal skin incision was located about 2 cm behind the mastoid process, with a length of about 5 cm (Figure 1).

3. The keyhole was next to the posterior groove of the digastric groove inferior to the junction of the transverse sinus and the sigmoid sinus. This is an oval bone window with a diameter of <2 cm (Figures 2, 3).

4. Cut the dura mater in a T shape and suspend the dura mater on the muscle tissue. The arachnoid was cut over the VII and VIII nerve complexes. The offending vessel was identified at the root entry zone. A small piece of Teflon sponge was then interposition between the brainstem and the artery to prevent the facial nerve from contacting the artery again.
After confirming the disappearance of the AMR wave, carefully suture the dura mater and suture the incision in layers.

### Clinical Evaluation

All patients were followed up by telephone or outpatient visits. The efficacy of surgery depends on the self-perception of the patient, mainly including the duration of HFS attacks and the daily frequency of attacks. The assessment of the offending vessels was determined through surgical records and surgical videos.

## Results

At surgical exploration, we found that the offending vessels may be a single blood vessel or multiple blood vessels working together. The most common offending vessels are the anterior inferior cerebellar artery (AICA) and the posterior inferior cerebellar artery (PICA). The offending vessels are in Table 2.

**Table 2:** Statistical results of the offending vessels.

**The offending vessels**	***n* (%)**
AICA	37 (25.0%)
PICA	48 (32.4%)
VA	8 (5.4%)
AICA + PICA	10 (6.8%)
AICA + VA	11 (7.4%)
PICA + VA	32 (21.6%)
AICA + PICA + VA	2 (1.4%)

*AICA, anterior inferior cerebellar artery; PICA, posterior inferior cerebellar artery; VA, vertebral artery*.

A grade of “cure” was assigned if HFS completely disappeared. A grade of “partial remission” was assigned if the spasm diminished by 75% or greater after the operation. We considered “cure” and “partial remission” to represent successful surgery. All other results were assigned a grade of “failure.” All grades were assessed within 24 h after micro-keyhole MVD.

Within 24 h after surgery, of the 148 patients, 120 (81.1%) exhibited complete relief (referred to as a cure), 24 (16.2%) expressed an improvement of more than 75% compared to pre-operative symptoms (referred to as partial remission), and four (2.7%) expressed an improvement of <75% or unchanged symptoms (referred as failure). During the follow-up period (more than 6 months), 14.1% (*n* = 23) of patients showed delayed cure. The detailed clinical outcomes after the operation (within 24 h after surgery) are shown in Table 3.

**Table 3:** Clinical outcomes of patients who underwent keyhole microvascular decompression (MVD) for hemifacial spasm (HFS).

**The offending vessels**	**Outcomes**		
	**Cure**	**Partial remission**	**Failure**
AICA	32	5	0
PICA	38	9	1
VA	8	0	0
AICA + PICA	9	1	0
AICA + VA	8	2	1
PICA + VA	23	7	2
AICA + PICA + VA	2	0	0
Total	120	24	4

*AICA, anterior inferior cerebellar artery; PICA, posterior inferior cerebellar artery; VA, vertebral artery*.

We defined post-operative facial nerve palsy (FNP) as grade II–VI according to the House-Brackmann scale (H-B scale) or any worsening of the grade if a patient had FNP before surgery. One patient (0.6%) exhibited iFNP (immediately FNP) and a total of 12 patients (8.1%) had dFNP (delayed PNP > 24 h).

We observed thirteen cases of facial nerve palsy seven cases of hearing loss (HL), and two cases of CSF leakage after surgery. There was one case with infarction, which was complicated by moyamoya disease.

All patients completed pure tone audiometry (PTA) before surgery. For patients complaining of HL, at 3–7 days after micro-keyhole MVD, a follow-up PTA was carried out by the otolaryngologist. We considered a meaningful HL as an increase of more than 15 dB of the average PTA (0.5, 1, and 2 K) threshold according to bone conduction (3).

We set the duration of follow-up to 6 months after surgery because the outcomes of MVD at 6 and 9 months showed similarities with those at >12 months (12). The major surgical complications (follow-up to 6 months) are shown in Table 4.

**Table 4:** Post-operative complications of patients who underwent keyhole MVD for HFS.

	**Cases, *n* (%)**
Hearing loss (transient, permanent)	7 (4.7%)
Transient HL	6 (4.1%)
Permanent HL	1 (0.6%)
Facial nerve palsy	13 (8.7%)
Immediate onset	1 (0.6%)
Delayed onset	12 (8.1%)
Symptoms related to CSF leakage	2 (1.3%)
Middle ear effusion	1 (0.6%)
CSF rhinorrhea	1 (0.6%)
Vascular complications	1 (0.6%)

*HL, hearing loss; CSF, cerebrospinal fluid*.

## Discussion

### Efficacy Analysis

Microvascular decompression is recognized as an effective treatment for HFS, and its effective rate is about 90% (2, 6, 13). In this series of cases, the effective rate of micro-keyhole MVD in the treatment of facial spasm was 97.2%, which was higher than that reported in much literature. During a 6-month post-operative follow-up, we observed 23(14.1%) patients presenting with delayed cure. The cases demonstrated that the micro-keyhole approach was effective for patients with HFS. Contrasting the endoscopic technique with the micro-keyhole MVD, there was no difference in the size of the bone window (all within 2 cm) (9, 10). Meantime, there was no difference in the effectiveness of MVD and the incidence of surgical complications (14).

By using a microscope, the size of the bone window of the MVD has been gradually reduced. Dr. Li believed a craniectomy of 3 cm in diameter was enough for most cases, which was based on the analysis of more than 4,000 cases (8). In the 2,040 patient series of Lee, a craniectomy with a size of 20 × 25mm was used (3). In the 197 patient series of Alford, the mean craniectomy size was about 33.8mm (15). However, to ensure the operation field of vision and avoid missing the responsible blood vessel, the size of the bone window has been limited to about 30mm. In this series, the bone window was designed to be <2 cm oval (Figures 2, 3). This size was significantly smaller than those reported in other literature. The effective rate of micro-keyhole MVD in the study was similar to those reported in the literature (3–6). We conclude that the key point of the micro-keyhole MVD lies in the placement of the burr hole instead of the size. Accurate placement of burr holes provides a convenient approach and adequate exposure. Compared with the traditional retrosigmoid keyhole approach, the location of the burr hole in the study was lower. A lower perspective avoids the cover of the acoustic nerve and provides direct exposure of the REZ of the facial nerve. The design of the surgical incision is the prerequisite for the design of the bone window. The traditional straight incision was used in this research. Chibbaro et al. described in detail an innovative C-shaped incision, which not only accurately exposes the bone window but also makes the surgical incision more minimally invasive (16).

The assumption of a poor outcome in patients with VA-involved HFS had been confirmed by some previous evidence (17, 18). However, with the sample, we observed a similar cure rate between the two patient populations during the 6 months of follow-up (*P* > 0.05), which is consistent with the results reported in the latest literature (19). We speculated that cover of vertebral artery easily leads to poor exposure of facial nerve and inadequate decompression. Therefore, we consider that when MRI indicates that the vertebral artery is possibly the responsible vessel, the REZ area should be thoroughly inspected to avoid missing any other responsible vessels. Usually, the PICA and/or its branches were pushed by the vertebral artery to compress the REZ of the facial nerve, which is a very common pattern of compression and needs to be noted.

Meantime, due to the small bone window, conventional connecting pieces can be used for skull reconstruction. Compared with the use of titanium mesh for skull reconstruction, this can save patients part of the treatment costs.

### Complications

Microvascular decompression, as a minimally invasive procedure, has been proven to be an effective treatment for HFS. However, complications of surgery can significantly affect patient satisfaction. In the present series of cases, micro-keyhole MVD not only decreased the incidence of CSF leakage-related symptoms and HL, but also did not increase the incidence of facial nerve paralysis or posterior cranial nerve injury.

### Cerebrospinal Fluid Leakage-Related Symptoms

We defined CSF leakage as post-operative CSF rhinorrhea and middle ear effusion. There was no middle ear effusion on the first review CT of the head after surgery, but the second review CT showed middle ear effusion, which was considered evidence of CSF leakage. The incidence of CSF leakage of 1.3% was observed in the study, which is less than the incidence of CSF leakage of 2.8–8.3% in patients undergoing MVD for HFS reported by other literature (2, 3, 15, 20, 21).

**Figure 4:**
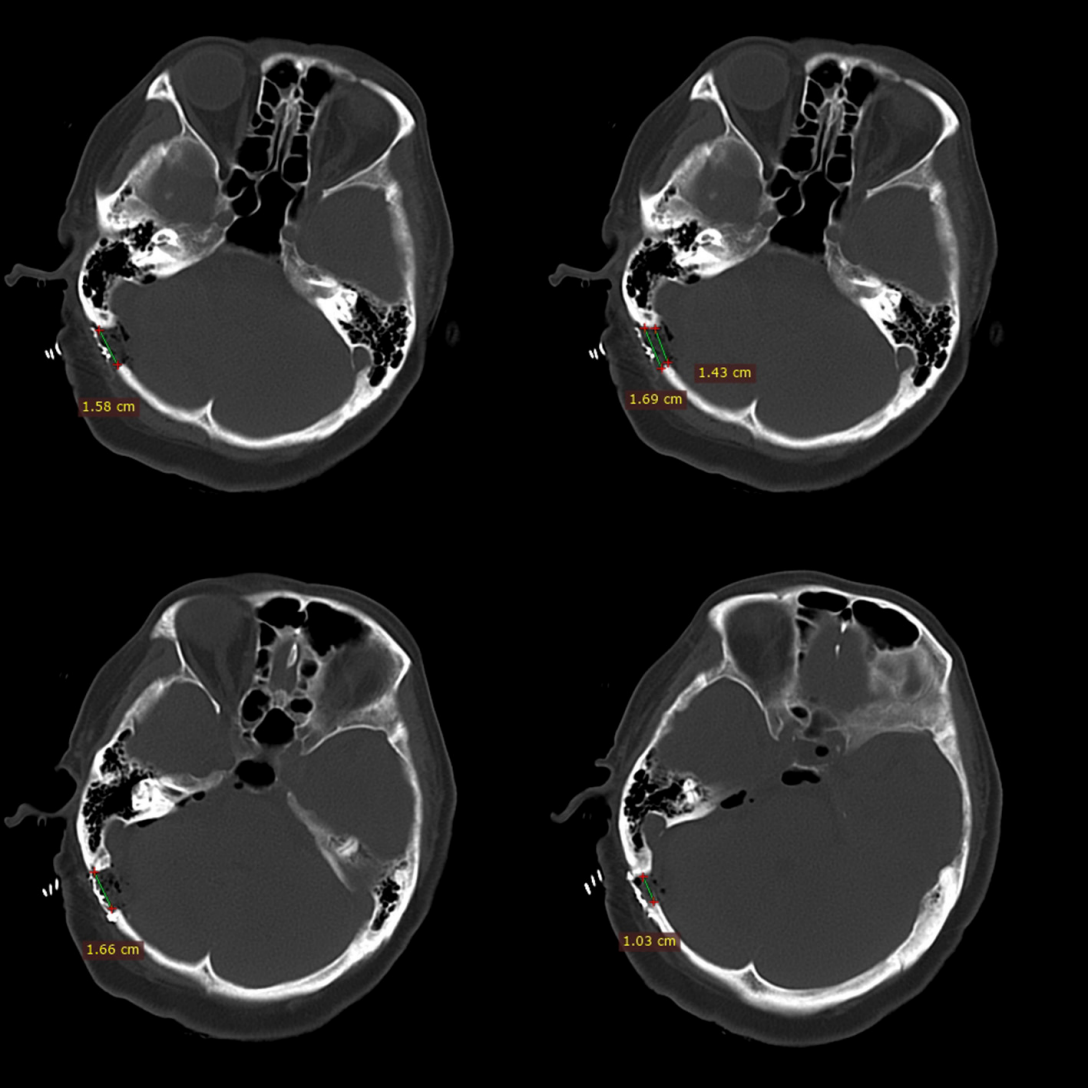
This figure shows the relative positional relationship between the bone window and the mastoid air chamber. Minimally invasive craniotomy (within 2 cm) can avoid opening the developed mastoid air chamber.

By designing a longitudinal direction, lower bone window, most of the mastoid air cells can be avoided to be opened. When the mastoid air cell is found to be opened during the operation, it should be thoroughly waxed, which can effectively avoid the occurrence of CSF leakage. The dura mater is suspended from the muscles when it is cut open, which helps prevent dural contracture and promotes tight dural closure. However, we believe that the smaller size of the bone window is more conducive to avoid opening the mastoid air cells. Especially for patients with well-developed mastoid air cells, it is better to avoid opening the mastoid cells, thereby reducing CSF leakage (Figure 4).

In reports of clear craniectomy size, the smaller the bone window, the lower the incidence of CSF leakage (3, 14, 15, 22). Compared with the series of cases, we speculated that the size of the bone window is negatively related to the incidence of CSF leakage, but this requires further exploration. Moreover, the keyhole approach means a small incision was sufficient for exposure. There was no incisional CSF leakage found in the study.

### Hearing Loss

Hearing loss after MVD for HFS can occur for the following reasons: stretching of cranial nerve VIII during cerebellar retraction, direct trauma to the nerve caused by instruments or nearby coagulation, outer hair cell dysfunction due to drill-induced noise, compromised blood supply, and bone dust deposit during drilling and fluid entering into the opened mastoid air cell (13, 23–25). In the present series, the incidence of HL was 4.7%, which was similar to the incidence of 2.7–12% reported in other literature (3, 4, 13, 26, 27), but the incidence of permanent HL (*n* = 1, 0.6%) was lower than that reported in other literature (21, 28). We speculated that it was related to the following factors: (1) a small and lower bone window could better avoid exposure to a mastoid air chamber. It reduced the occurrence of conductive HL by reducing the occurrence of fluid entering into the opened mastoid air cell (23). (2) using the drill to make only a hole and completing the craniectomy by hand avoiding drill-induced noise damage to high-frequency hearing (29, 30). (3) For cases of macroscopic vasospasm during the operation, we used papaverine directly on the vessel wall to manage vasospasm after decompression.

### Facial Nerve Palsy

The incidence of dFNP paralysis has been reported to be from 2.8 to 22.5% (3, 4, 27, 31, 32), which is similar to the incidence of dFNP of 8.1% observed in the study. Possible causes of delayed facial paralysis include facial nerve exit zone injury *via* the Teflon felt, delayed facial nerve edema, activation of dormant viruses, or microcirculation disturbance due to vasospasm. The result was similar to previous literature reports. All of the patients who experienced dFNP were clinically cured during follow-up.

iFNP is considered to be an incurable surgical complication, which is generally believed to be related to the direct damage of the facial nerve to the post-operative operation (4). The number of incidences of iFNP in the study was 0.6%, which was similar to other studies (2, 4, 27, 33). Therefore, we consider that the small bone window does not increase the difficulty of visual field exposure and the risk of direct damage to the facial nerve.

### Strokes and Hematomas

In the present series of cases, one patient had an acerebral infarction. This patient was diagnosed with moyamoya disease by MR angiography. We considered that cerebral infarction was related to lower blood pressure during surgery. This also reminds us that it is meaningful for patients to undergo head angiography before MVD surgery.

Through retrospective analysis of post-operative head CT, no obvious cerebellar contusion, cerebellar hemorrhage, and other complications were found. Unfortunately, post-operative head MRI had not been used to assess the related invasive injuries. We believe that by rationally designing the position and shape of the bone window, the micro-keyhole will not increase excessive stretching on cerebellar tissue.

### Limitations

There are several limitations to this study. This is a single-center retrospective evaluation at an institution; the safety and effectiveness of the operation may be affected by the experience of the surgeon. We are trying to cooperate with other medical centers to verify the effectiveness and repeatability of micro-keyhole MVD. Since the sample size was determined by the number of patients in the inclusion period, the sample size was small. Further research may consider assessing the rapid post-operative recovery and subjective feeling of patients involved in a smaller bone window, to evaluate whether minimally invasive surgery can provide patients with a better medical experience.

## Conclusion

We reviewed 148 patients who underwent micro-keyhole MVD for HFS. The micro-keyhole MVD technique has no significant difference in the cure rate and the incidence of surgical complications compared with the larger bone window. At the same time, the smaller bone window reduces the incidence of CSF leakage and HL. In general, the <2 cm micro-keyhole MVD with a more minimally invasive bone window is a safe and effective surgical method to treat HFS.

## Data Availability Statement

The original contributions presented in the study are included in the article/supplementary material, further inquiries can be directed to the corresponding author/s.

## Ethics Statement

Written informed consent was obtained from the individual(s) for the publication of any potentially identifiable images or data included in this article.

## Author Contributions

This study was designed and managed by JH and YZ, with data collected and processed by JH, LJ, and YL. Data were analyzed by JH and YL. The manuscript was prepared by JH, YL, LJ, KW, ZW, QS, YX, and YZ. All authors contributed to the article and approved the submitted version.

## Conflict of Interest

The authors declare that the research was conducted in the absence of any commercial or financial relationships that could be construed as a potential conflict of interest.

## Publisher's Note

All claims expressed in this article are solely those of the authors and do not necessarily represent those of their affiliated organizations, or those of the publisher, the editors and the reviewers. Any product that may be evaluated in this article, or claim that may be made by its manufacturer, is not guaranteed or endorsed by the publisher.

## References

[1] Jannetta PJ Abbasy M Maroon JC Ramos FM Albin MS. Etiology and definitive microsurgical treatment of hemifacial spasm. Operative techniques and results in 47 patients. J Neurosurg. (1977) 47:321–8. 10.3171/jns.1977.47.3.0321 894338

[2] Sindou M Mercier P. Microvascular decompression for hemifacial spasm: outcome on spasm and complications. A review. Neurochirurgie. (2018) 64:106–16. 10.1016/j.neuchi.2018.01.001 29454467

[3] Lee MH Jee TK Lee JA Park K. Postoperative complications of microvascular decompression for hemifacial spasm: lessons from experience of 2040 cases. Neurosurg Rev. (2016) 39:151–8; discussion 158. 10.1007/s10143-015-0666-7 26382646

[4] Zhao H Zhang X Tang YD Zhang Y Ying TT Zhu J. Operative complications of microvascular decompression for hemifacial spasm: experience of 1548 cases. World Neurosurg. (2017) 107:559–64. 10.1016/j.wneu.2017.08.028 28823667

[5] Jo KW Kong DS Park K. Microvascular decompression for hemifacial spasm: long-term outcome and prognostic factors, with emphasis on delayed cure. Neurosurg Rev. (2013) 36:297–301; discussion 301-2. 10.1007/s10143-012-0420-3 22940822

[6] Holste K Sahyouni R Teton Z Chan AY Englot DJ Rolston JD. Spasm freedom following microvascular decompression for hemifacial spasm: systematic review and meta-analysis. World Neurosurg. (2020) 139:e383–90. 10.1016/j.wneu.2020.04.001 32305605 PMC7899163

[7] McLaughlin MR Jannetta PJ Clyde BL Subach BR Comey CH Resnick DK. Microvascular decompression of cranial nerves: lessons learned after 4400 operations. J Neurosurg. (1999) 90:1–8. 10.3171/jns.1999.90.1.0001 10413149

[8] Zhong J Zhu J Sun H Dou NN Wang YN Ying TT. Microvascular decompression surgery: surgical principles and technical nuances based on 4000 cases. Neurol Res. (2014) 36:882–93. 10.1179/1743132814Y.0000000344 24597913

[9] Charalampaki P Kafadar AM Grunert P Ayyad A Perneczky A. Vascular decompression of trigeminal and facial nerves in the posterior fossa under endoscope-assisted keyhole conditions. Skull Base. (2008) 18:117–28. 10.1055/s-2007-1003927 18769532 PMC2435473

[10] Zhu J Sun J Li R Yu Y Zhang L. Fully endoscopic versus microscopic vascular decompression for hemifacial spasm: a retrospective cohort study. Acta Neurochir (Wien). (2021). 10.1007/s00701-021-04824-0. [Epub ahead of print]. 33765219

[11] Feng BH Zhong WX Li ST Wang XH. Fully endoscopic microvascular decompression of the hemifacial spasm: our experience. Acta Neurochir (Wien). (2020) 162:1081–7. 10.1007/s00701-020-04245-5 32133573

[12] Lee JA Park K. Short-term versus long-term outcomes of microvascular decompression for hemifacial spasm. Acta Neurochir. (2019) 161:2027–33. 10.1007/s00701-019-04032-x 31392569

[13] Miller LE Miller VM. Safety and effectiveness of microvascular decompression for treatment of hemifacial spasm: a systematic review. Br J Neurosurg. (2012) 26:438–44. 10.3109/02688697.2011.641613 22168965

[14] Li Y Mao F Cheng F Peng C Guo D Wang B. A meta-analysis of endoscopic microvascular decompression versus microscopic microvascular decompression for the treatment for cranial nerve syndrome caused by vascular compression. World Neurosurg. (2019) 126:647–55.e647. 10.1016/j.wneu.2019.01.220 30776512

[15] Alford EN Chagoya G Elsayed GA Bernstock JD Bentley JN Romeo A. Risk factors for wound-related complications after microvascular decompression. Neurosurg Rev. (2020) 44:1093–101. 10.1007/s10143-020-01296-1 32306156

[16] Chibbaro S Cebula H Scibilia A Spatola G Todeschi J Gubian A. Retrosigmoid approach: investigating the role of a C-shaped skin incision and muscle flaps in improving functional outcome and reducing postoperative pain. World Neurosurg. (2018) 111:e340–7. 10.1016/j.wneu.2017.12.050 29258939

[17] Kim JP Park BJ Choi SK Rhee BA Lim YJ. Microvascular decompression for hemifacial spasm associated with vertebrobasilar artery. J Korean Neurosurg Soc. (2008) 44:131–5. 10.3340/jkns.2008.44.3.131 19096662 PMC2588300

[18] Jiang C Liang W Wang J Dai Y Jin W Sun X. Microvascular decompression for hemifacial spasm associated with distinct offending vessels: a retrospective clinical study. Clin Neurol Neurosurg. (2020) 194:105876. 10.1016/j.clineuro.2020.105876 32413816

[19] Lee S Han J Park SK Lee JA Joo BE Park K. Involvement of the vertebral artery in hemifacial spasm: clinical features and surgical strategy. Sci Rep. (2021) 11:4915. 10.1038/s41598-021-84347-x 33649393 PMC7921589

[20] Khan SA Laulloo A Vats A Nath F. Microvascular decompression: incidence and prevention of postoperative CSF leakage in a consecutive series of 134 patients. Br J Neurosurg. (2020) 34:416–8. 10.1080/02688697.2020.1749989 32362141

[21] Sharma R Garg K Agarwal S Agarwal D Chandra PS Kale SS. Microvascular decompression for hemifacial spasm: a systematic review of vascular pathology, long term treatment efficacy and safety. Neurol India. (2017) 65:493–505. 10.4103/neuroindia.NI_1166_16 28488609

[22] Ma Z Li M Cao Y Chen X. Keyhole microsurgery for trigeminal neuralgia, hemifacial spasm and glossopharyngeal neuralgia. Eur Arch Otorhinolaryngol. (2010) 267:449–54. 10.1007/s00405-009-1005-9 19536556

[23] Ying T Thirumala P Gardner P Habeych M Crammond D Balzer J. The incidence of early postoperative conductive hearing loss after microvascular decompression of hemifacial spasm. J Neurol Surg B Skull Base. (2015) 76:411–5. 10.1055/s-0034-1390402 26682118 PMC4671886

[24] Jo KW Lee JA Park K Cho YS. A new possible mechanism of hearing loss after microvascular decompression for hemifacial spasm. Otol Neurotol. (2013) 34:1247–52. 10.1097/MAO.0b013e31829b5786 23942352

[25] Ying T Thirumala P Shah A Nikonow T Wichman K Holmes M. Incidence of high-frequency hearing loss after microvascular decompression for hemifacial spasm. J Neurosurg. (2013) 118:719–24. 10.3171/2013.1.JNS121153 23394342

[26] Shah A Nikonow T Thirumala P Hirsch B Chang Y Gardner P. Hearing outcomes following microvascular decompression for hemifacial spasm. Clin Neurol Neurosurg. (2012) 114:673–77. 10.1016/j.clineuro.2012.01.016 22410649

[27] Huh R Han IB Moon JY Chang JW Chung SS. Microvascular decompression for hemifacial spasm: analyses of operative complications in 1582 consecutive patients. Surg Neurol. (2008) 69:153–7; discussion 157. 10.1016/j.surneu.2007.07.027 18261641

[28] Bartindale M Kircher M Adams W Balasubramanian N Liles J Bell J. Hearing loss following posterior fossa microvascular decompression: a systematic review. Otolaryngol Head Neck Surg. (2018) 158:62–75. 10.1177/0194599817728878 28895459 PMC7147641

[29] Zou J Bretlau P Pyykkö I Starck J Toppila E. Sensorineural hearing loss after vibration: an animal model for evaluating prevention and treatment of inner ear hearing loss. Acta Otolaryngol. (2001) 121:143–8. 10.1080/000164801300043244 11349766

[30] Yin X Strömberg AK Duan M. Evaluation of the noise generated by otological electrical drills and suction during cadaver surgery. Acta Otolaryngol. (2011) 131:1132–5. 10.3109/00016489.2011.600725 21756022

[31] Soriano-Baron H Vales-Hidalgo O Arvizu-Saldana E Moreno-Jimenez S Revuelta-Gutierrez R. Hemifacial spasm: 20-year surgical experience, lesson learned. Surg Neurol Int. (2015) 6:83. 10.4103/2152-7806.157443 26015871 PMC4443403

[32] Amagasaki K Kurita N Watanabe S Shono N Hosono A Naemura K. Lower cranial nerve palsy after the infrafloccular approach in microvascular decompression for hemifacial spasm. Surg Neurol Int. (2017) 8:67. 10.4103/sni.sni_8_17 28540133 PMC5421220

[33] Dannenbaum M Lega BC Suki D Harper RL Yoshor D. Microvascular decompression for hemifacial spasm: long-term results from 114 operations performed without neurophysiological monitoring. J Neurosurg. (2008) 109:410–5. 10.3171/JNS/2008/109/9/0410 18759569

